# Euthanasia of a macaque with self-injurious behavior in a visual neuroscience laboratory

**DOI:** 10.48130/animadv-0026-0018

**Published:** 2026-07-28

**Authors:** Jonathan C. Horton

**Affiliations:** Program in Neuroscience, University of California, San Francisco, San Francisco, California, 94143, USA

**Keywords:** Euthanasia, Pair-housing, Animal welfare, Environmental enrichment, Non-human primate

## Abstract

The causes of self-injurious behavior (SIB) in primates are not well understood, but a relatively impoverished physical environment, deficiency of normal social interactions, lack of novel stimulation, nursery rather than maternal rearing, and an intrinsically susceptible temperament are potential contributing factors. An important issue, seldom addressed, is what level of SIB is severe enough to warrant euthanasia. In this case report, a healthy 5-year-old macaque is described who was euthanized 11 d after developing SIB. The episodes occurred about 0.5% of the time and were rated SIB 1 (mild) per the United States National Primate Research Center Scoring System. The factors that may have led to the emergence of SIB, the efforts made to address it, and the decision to euthanize the monkey are reviewed. Recommendations are made for management of future cases, along with a suggestion that self-injurious behavior should conform to category SIB 2 (moderate) or SIB 3 (severe) before euthanasia is considered.

## Introduction

Self-injurious behavior (SIB) exhibited by macaques housed in research facilities is a serious problem^[1–3]^. It occurs among 5%–25% of animals at primate centers in the United States^[4–7]^. The wide range in reported prevalence is due partly to differences in ascertainment method, housing conditions, rearing history, age, sex, and the definition of SIB employed by investigators. The latter is a particularly important consideration. Monkeys may place an ankle or wrist in their mouth and appear to bite, but no wound is visible on subsequent examination. Under such circumstances, the potential for injury from SIB exists, but none has actually occurred. Novak and colleagues surveyed 362 macaques at a National Primate Research Center and observed that 25% of the monkeys engaged in self-directed biting, but only 11% had a self-inflicted wound detectable by veterinary examination^[8]^. Even among the 11% of animals with verified SIB, wounding was a rare event, with a median of only two lifetime episodes recorded per animal.

To define conduct that constitutes SIB more precisely, the United States National Primate Research Centers have developed a scoring system containing five categories (https://nprcresearch.org/research/page/BMC_-_Self-injurious_Behavior_Scoring_System). The first two categories are assigned if no visible wound has resulted from the animal's behavior. They are used to describe self-biting (Category 1) or other potentially harmful non-biting behaviors, such as head banging or hair plucking (Category 2). Mild SIB refers to superficial wounds that do not require medical attention, such as bruising, small punctures, and pinpoint lesions (Category 3). Moderate SIB involves superficial lacerations and punctures that may require veterinary attention (Category 4). Severe SIB is represented by deep, large lacerations or punctures that require suturing, amputation, or other treatments (Category 5). This self-injurious behavior scoring system is potentially somewhat confusing because the first two categories refer to behaviors that do not actually result in visible self-injury.

One practical problem with the implementation of this SIB rating system is that it can be difficult to differentiate between Category 1 (involving no actual physical harm) and Category 3, because minor bruising or pinpoint excoriation is hard to detect in a monkey moving freely within its enclosure. The guidelines provide no recommendation regarding the advisability of shaving the fur covering the wrist or ankle to make it easier to monitor these sites for superficial skin injury.

In addition to defining the severity of SIB using this five-category system, it is important to quantify the frequency of SIB. Video recording is extremely useful for this purpose because it allows systematic collection of data over long periods, avoiding reliance on briefer, anecdotal observations^[9,10]^. One can measure precisely the occurrence rate of SIB, detect infrequent events, identify triggering factors, and measure the impact of any mitigatory interventions. Video recording also avoids the presence of a human spectator in the room, which has been shown to affect the occurrence rate of SIB^[6]^.

Over the past 23 years, our laboratory has conducted research on strabismus using a macaque model^[11]^. Strabismus is a common condition that affects millions, causing loss of stereopsis and, in some cases, reduced vision from amblyopia. The disease cannot be investigated in rodents because they lack a fovea or area centralis. To understand the underlying neural mechanism, we have performed electrophysiological recordings in alert macaques raised with strabismus induced during infancy^[12,13]^.

The monkeys used for our research were housed in a small vivarium located adjacent to the laboratory where daily recording sessions were performed. Several years ago, an animal began to manifest SIB. The monkey was euthanized 11 days later. The goals of this case report are to analyze the factors that may have led to the emergence of SIB and to provide practical guidance pertaining to measures that might have been taken to avoid euthanasia. A related issue is to address what level of SIB reaches a threshold at which euthanasia may be justified as an appropriate measure. There is an extensive literature pertaining to SIB in macaques, but few articles address this difficult question^[14,15]^.

## Methods

The animals described in this report were used in accordance with a protocol and housed in a vivarium approved by the Institutional Animal Care and Use Committee (IACUC). The vivarium contained two Rhesus (*Macaca mulatta*) monkeys, named Clive and Jude, both born and raised at the California National Primate Research Center. Clive was a 13-year-old male with exotropia weighing 14.8 kg who had participated for many years in single-cell recording experiments. Jude was a normal 5-year-old male weighing 16.4 kg who was ready to begin chair training in preparation for a research project. He had been living in the same room with Clive for just over a year. The circumstances surrounding the emergence of SIB in Jude are described. Episodes of SIB were quantified by video recording. The category of SIB was defined by direct behavioral observations, examinations under anesthesia, and post-mortem examination. Case information was based on the animal's medical records, video recordings, e-mail exchanges, and contemporarily recorded notes from meetings held to review the animal's behavior.

## Results

The two animals were housed in a caging system manufactured by Primate Products, Inc., configured to give them approximately twice the floor space and vertical height recommended for Group 4 monkeys, National Research^[16]^. Each animal lived in a home module with a floor measuring 6 square feet and a height of 32 inches. The modules were arranged in a stacked over/under configuration. From his home module, each monkey could freely enter his own adjacent activity module with a floor measuring 6 square feet and a height of 72 inches. Both the home module and the activity module contained a broad shelf for perching. The home modules were equipped with a squeeze mechanism. Monkeys could be confined temporarily to their home module if necessary by closing a set of sliding polycarbonate panels that served as side doors between each module.

**Fig. 1:**
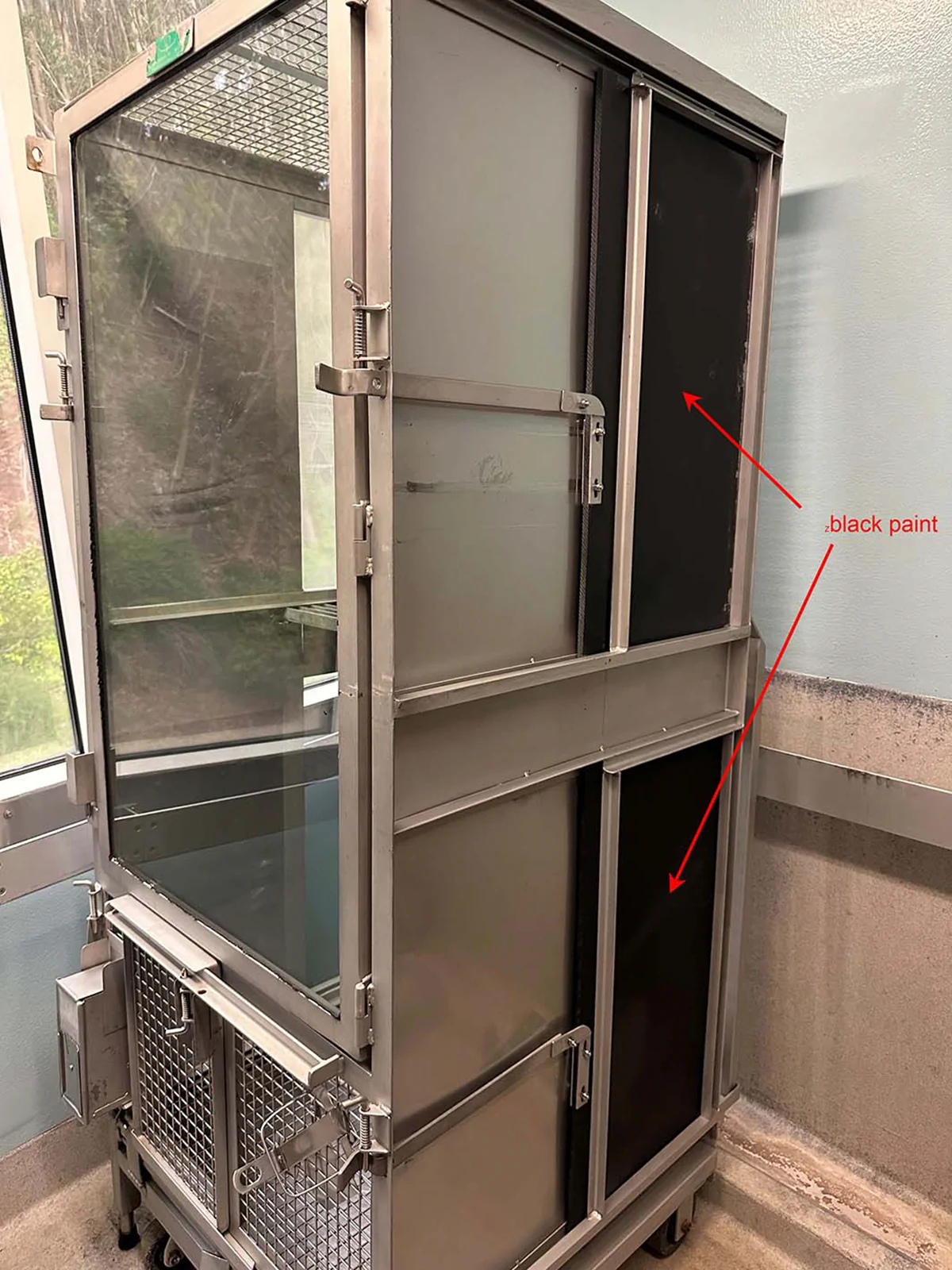
The activity module occupied by Clive, showing the sliding panels that served as side doors. They were painted black to prevent visual interaction with a monkey in an adjacent home module with whom Clive was hostile. After that monkey was permanently removed from the vivarium, the panels were left opaque to prevent visual contact between Clive and Jude.

The transparent polycarbonate panels on Clive's modules were painted black to prevent visual contact with any monkey in an adjacent module. This was done because 5 years previously, while performing a cage rotation, the husbandry staff inadvertently left open the sliding panels separating Clive from another monkey. The two animals immediately began fighting. Before they could be separated, both suffered multiple lacerations that required suture repair under general anesthesia. Following this episode, the polycarbonate panels were rendered opaque in the hope that eliminating visual contact between Clive and other monkeys would reduce aggressive behavior and mitigate stress being experienced by the animals (Fig. 1).

Shortly before Jude began to exhibit SIB, a third monkey lived in the vivarium. Electrophysiological recordings had been made from this animal over many months, followed by a terminal anatomical experiment. All three monkeys were housed individually in home modules and had access at different times to a single activity module. Jude and the third monkey had direct visual contact through transparent panels separating their adjacent modules.

Immediately after the third monkey was removed, one of the two over/under home module units in the vivarium room was exchanged for an activity module, so that Jude and Clive each had exclusive, full-time use of an activity module. The veterinary staff also conducted an assessment of Clive and Jude's compatibility, observed hostile interactions, and concluded that they could not be pair-housed safely. For both animals' environmental enrichment, the veterinary technicians provided three daily 15-min training sessions involving positive reinforcement for presentation of an arm or leg, plus a wide variety of foraging devices.

**Fig. 2:**
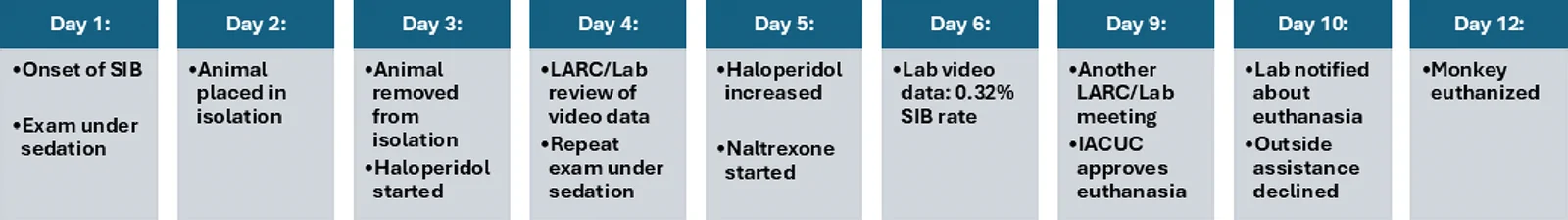
Timeline of events from day 1 when SIB was first observed to euthanasia 11 days later.

Nine days after the third monkey was removed from the room, Jude was observed to be placing an ankle in his mouth and biting it (Fig. 2). He was immediately sedated for examination, which revealed slight bruising but no lacerations or punctures. The next day, veterinary staff decided to move him to a separate room where he was kept alone, in the hope that isolation from Clive would stop the SIB. During 24 h of solitary housing, his SIB continued, so he was returned to his original quarters. Treatment was started with oral haloperidol 0.5 mg twice per day.

Three days after the onset of SIB, a meeting was held between our laboratory and the Laboratory Animal Resource Center (LARC) staff. They had recorded many hours of video to document Jude's behavior. They reported that analysis of the video showed an SIB occurrence rate of about one episode per min. However, when a 15-min video segment was screened together, no episodes were observed. Fast-forwarding through other video files confirmed the existence of SIB episodes. The occurrence rate in each video file was not specified. The lab requested to view the video data independently to quantify the occurrence rate. The LARC leadership advised that the video data could not be shared and, in fact, would be deleted after the meeting.

**Fig. 3:**
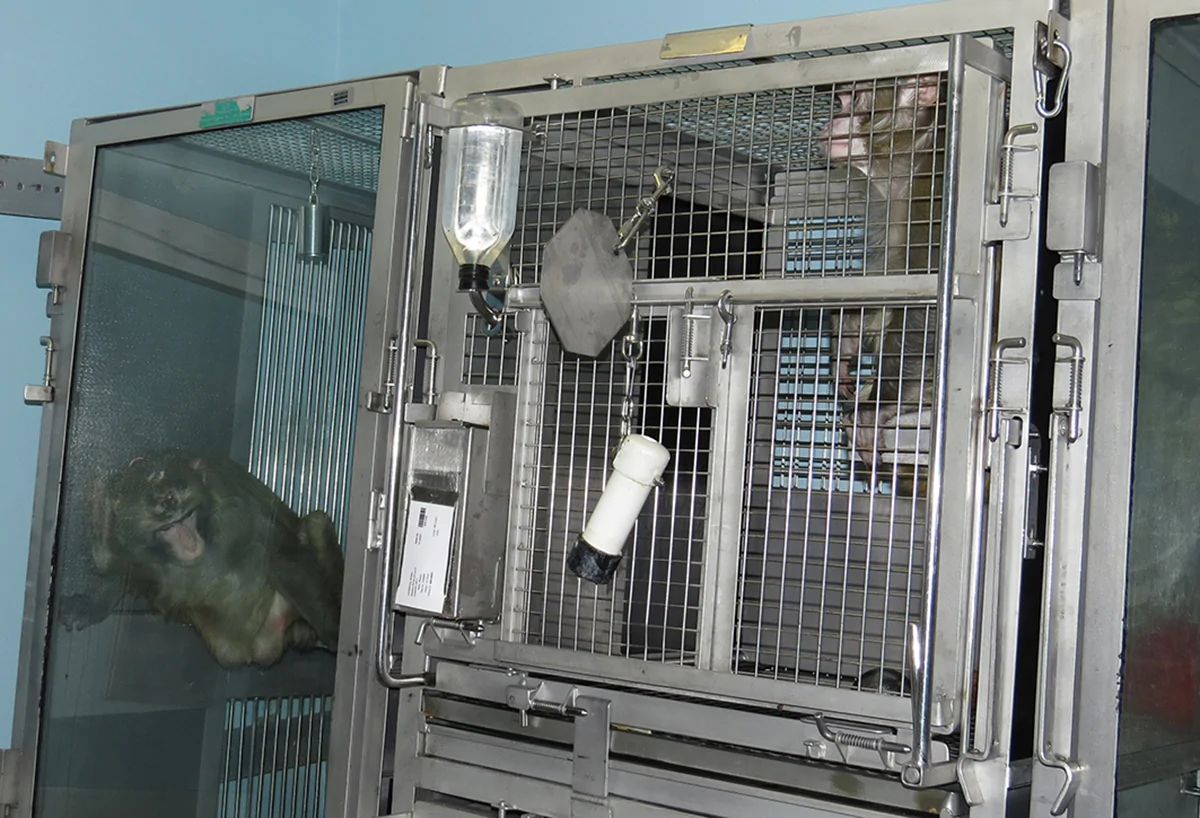
Clive in his activity module (left) and Jude in his home module (right) were unable to see each other, although they often positioned themselves against the front of their enclosures, apparently straining for a glimpse.

We requested removal of the black paint obscuring the panels on Clive's exercise module so that the two monkeys could have direct visual contact (Fig. 3). We also requested that Jude's limbs be shaved to facilitate monitoring for injury. These requests were declined, but the monkey was sedated later the same day for examination, which showed trace bruising of his lower extremities.

**Fig. 4:**
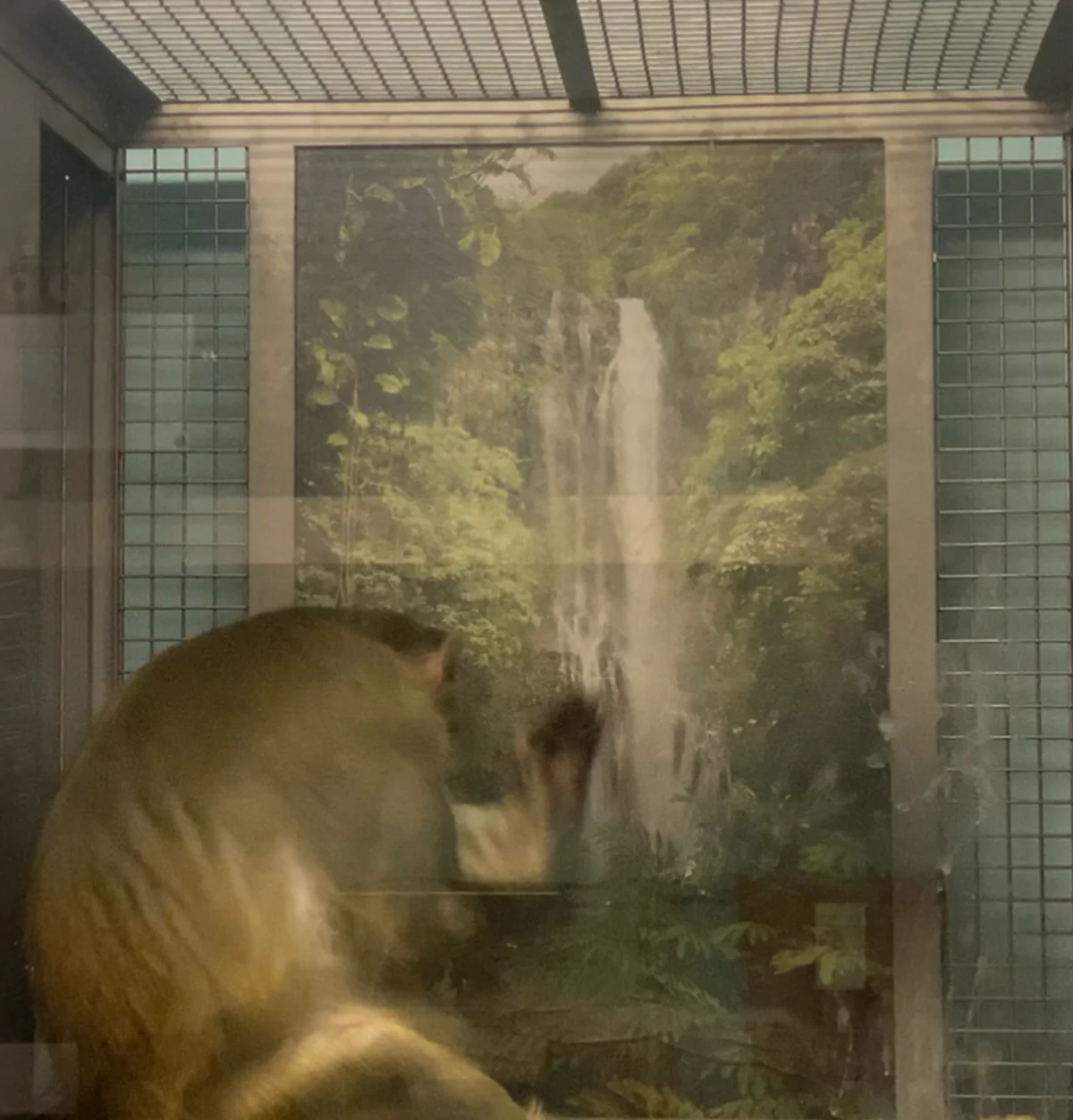
Jude engaging in SIB by grasping his left leg with his hands and placing it into his mouth. Rarely, he placed a wrist in this mouth.

Four days after the onset of SIB, the dosage of oral haloperidol was increased to 1.0 mg twice per day. Treatment was also initiated with oral naltrexone 50 mg once per day. The medications were crushed and hidden within jelly and grapes. Jude was a fastidious monkey with a habit of examining treats carefully. For example, when given a grape, he would spit out the skin. At the end of each day, we observed medication-laced treats in the waste collection pan under his cage, where he had dropped them. We requested that LARC convert from oral to intramuscular administration of medications, but they continued to be offered secreted in treats. The animal's SIB persisted (Fig. 4).

Five days after the onset of SIB we made video recordings of Jude's behavior. A representative 56-min recording showed a single episode of SIB lasting 11 s, corresponding to an occurrence rate of 0.32% (https://osf.io/4gkz8/files/osfstorage/68b7782265113fae162c2615).

Eight days after the onset of SIB, another meeting was held between the laboratory and LARC. We suggested consulting the veterinarians at the United States Department of Agriculture (USDA) for advice. The LARC leadership declined because of potential adverse repercussions. Later that day, LARC leadership met with the IACUC and presented a plan to euthanize the monkey.

The next day, it was announced that the monkey would be euthanized in 2 days. The director of a National Primate Research Center offered the services of an expert in primate behavior management to assess Jude and make recommendations. The assistance was declined.

The LARC leadership provided this rationale for euthanasia: 'I agree the animal's behavior is at level one [Category 3] based on the scale you sent. My goal is to ensure that this animal does not progress to level 2 or 3 [Category 4 or 5]. The LARC veterinarians have consulted with national primate center veterinarians and a number of experts in non-human primate behavior. All have agreed that this case is severe due to the frequency of the behavior. He has shown over 50 SIB incidents in a day'.

**Fig. 5:**
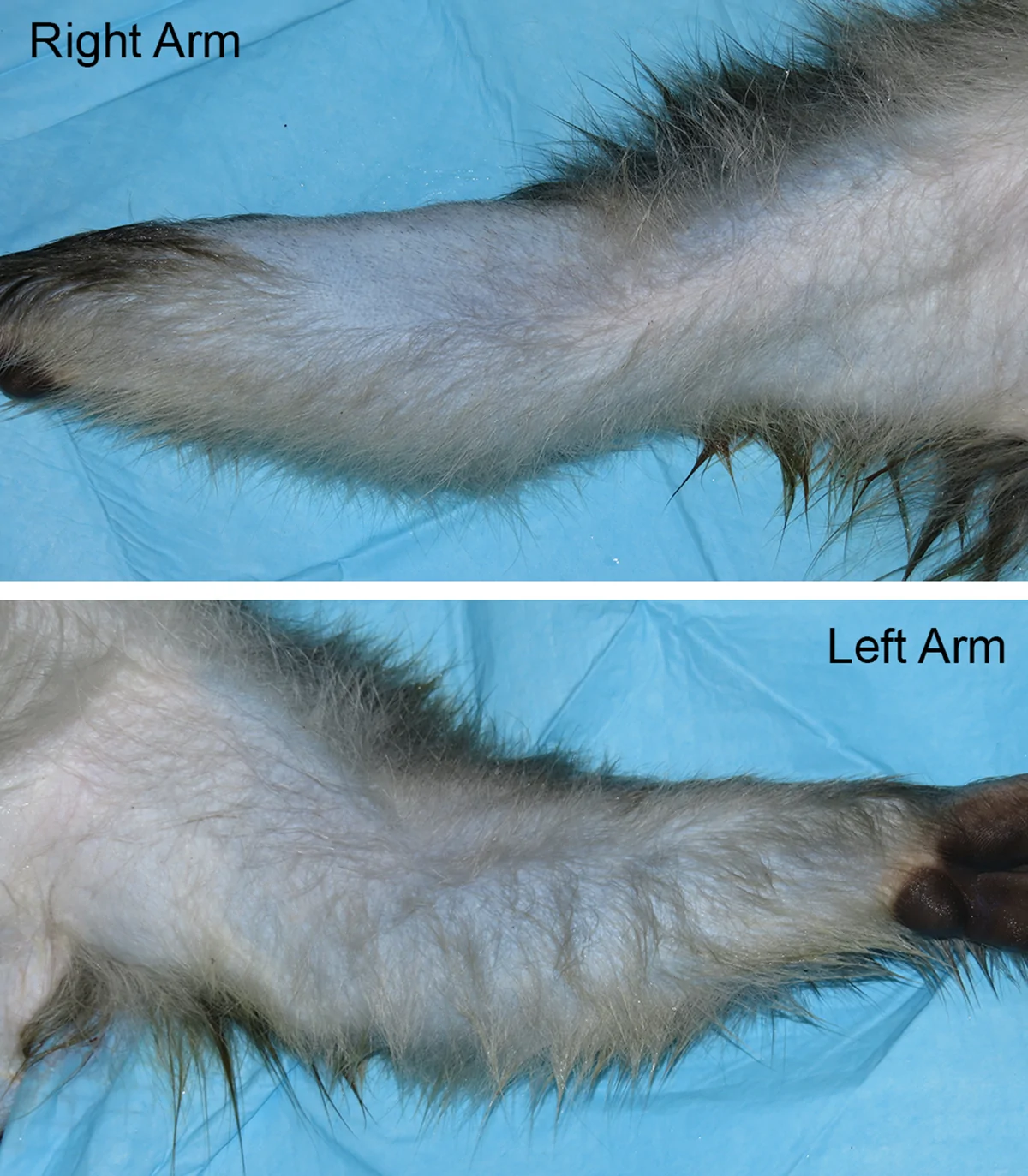
Post-mortem pictures of Jude showing no injuries to his arms.

**Fig. 6:**
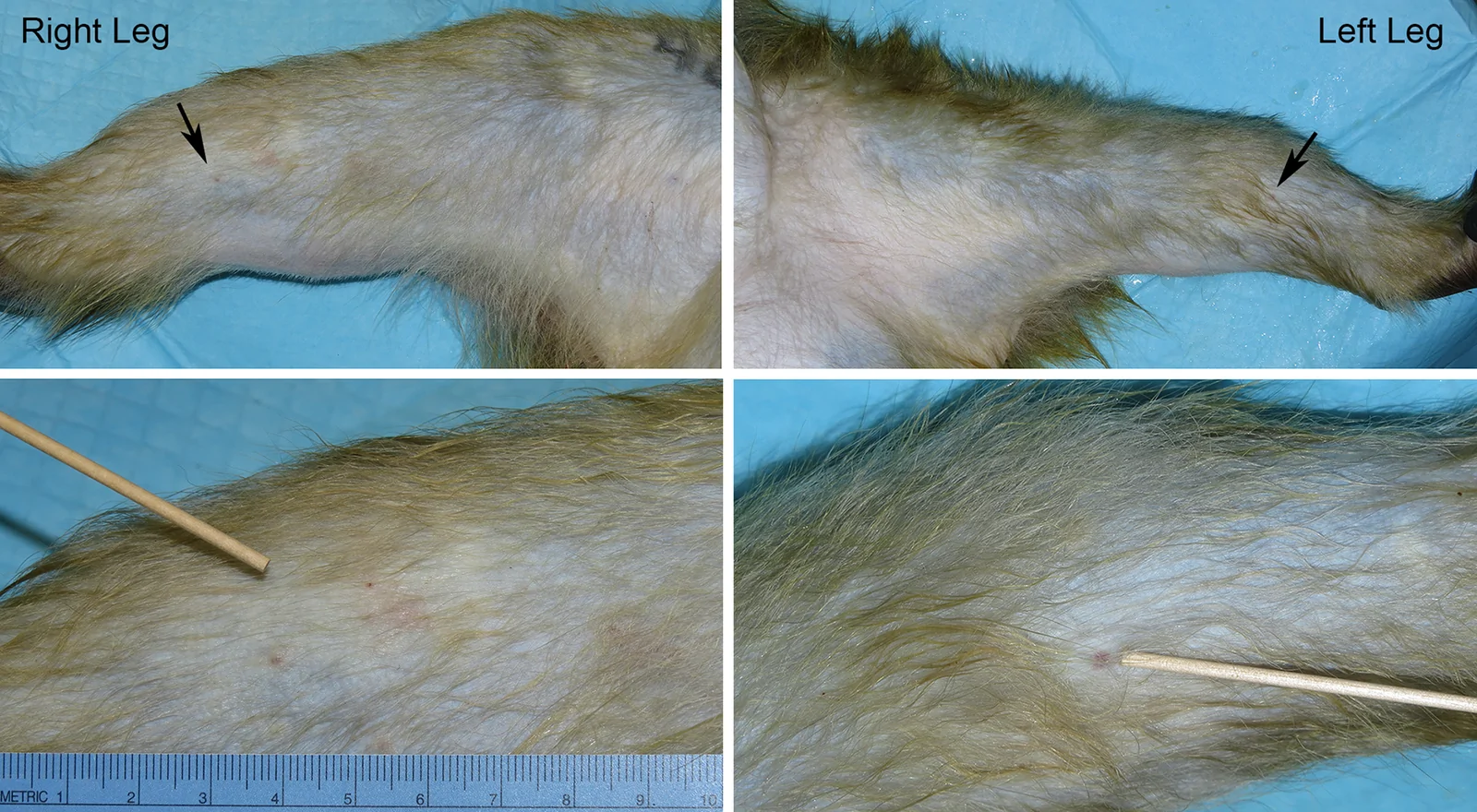
Post-mortem pictures of Jude showing mild, Category 3 SIB to his legs. (Top) Low power views, with arrows showing bruising and pinpoint scabs. (Bottom) High power views, with wooden applicator pointing to lesions.

Eleven days after the onset of SIB, the monkey was euthanized. Post-mortem examination showed no evidence of injury to the arms (Fig. 5). The left leg had a single ecchymosis with a diameter of 3 mm. The right leg had several scabs, each 1 mm in diameter, and a few faint bruises (Fig. 6).

## Discussion

SIB is disturbing to witness because it appears unnatural and violent, evoking a strong emotional reaction in the observer and a valid concern that the animal is suffering. It bears a striking resemblance to self-injurious behavior in humans, such as wrist cutting^[17–21]^. The motivation for SIB in monkeys and humans is unclear, but the act may actually bring relief to a subject by releasing psychic tension experienced from an escalating paroxysm of mental distress^[5,18,22,23]^.

To eliminate SIB, efforts should focus on identifying and mitigating the conditions responsible for it. It could have been coincidental, but it was notable that Jude began to manifest SIB only 9 d after a third monkey was removed from the vivarium. Previously, Jude had unlimited visual contact with this monkey and interacted with him, despite being housed separately. After a behavioral assessment, it was concluded that the remaining two monkeys in the vivarium—Jude and Clive—were incompatible for pair-housing. Due to concerns that aggressive posturing might ensue, they were isolated visually from each other by keeping the divider panels painted black between their modules. The National Research Council's guide (1998) to *The Psychological Well-Being of Nonhuman Primates* states that "Primates that continually threaten each other should be moved out of direct visual contact". However, it is not clear that hostile posturing between adult male monkeys is necessarily harmful to their mental health. Moreover, Clive and Jude had no history of threatening each other because they had never had visual contact, except for brief moments when Clive was transferring to a primate chair. A trial of visual exposure with Clive might have reduced Jude's SIB by alleviating his isolation and boredom.

**Fig. 7:**
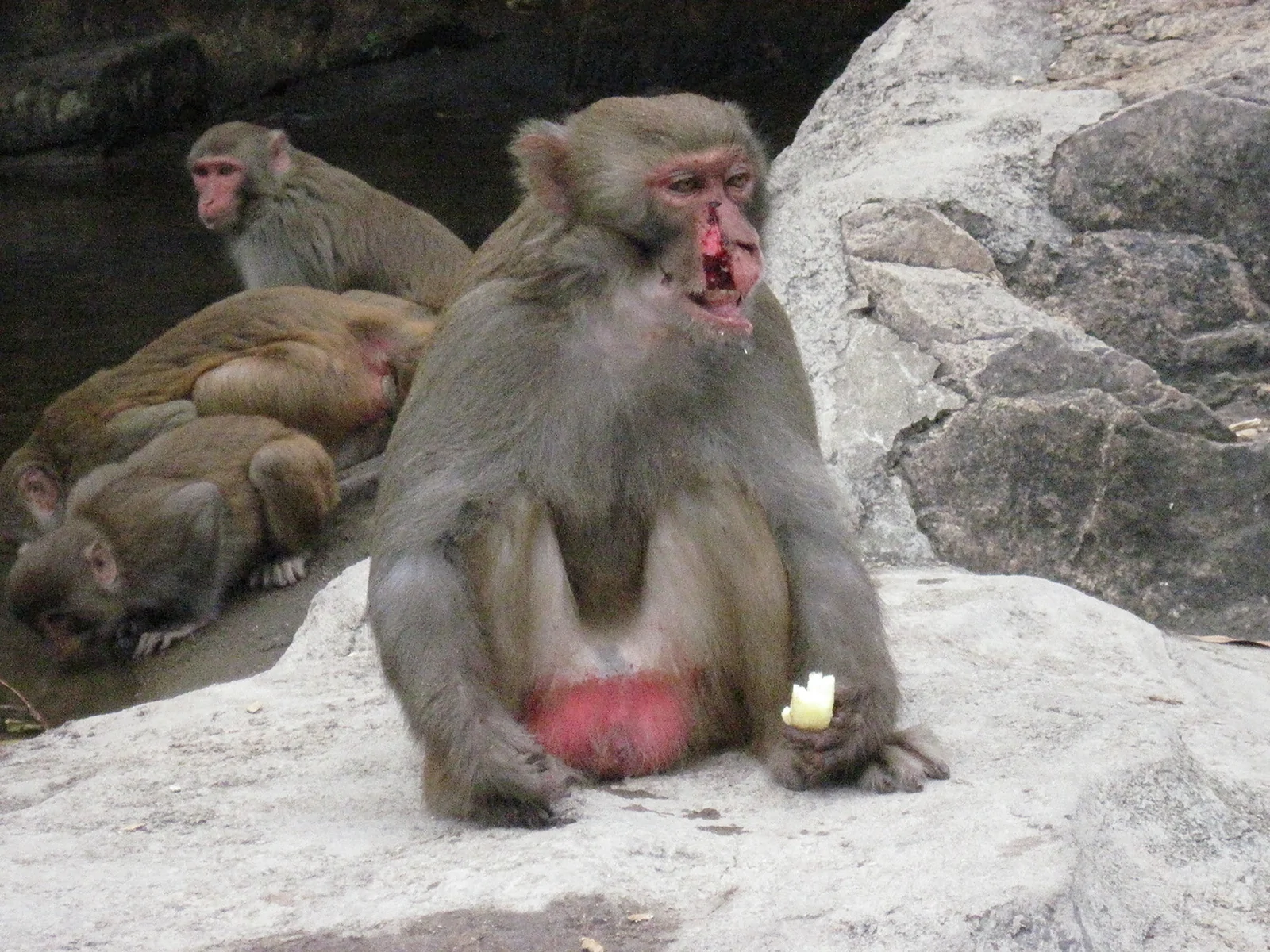
Free-ranging male Rhesus in a nature preserve with an 8 cm laceration exposing the maxillary bone from combat with another monkey.

A program of environmental enrichment was provided to Jude by veterinary technicians. Evidence is conflicting, but most studies have shown that such measures do not curb SIB effectively^[24–27]^. The most impactful measure to reduce SIB among research monkeys is to house them together^[28–31]^. In recent years, there has been a laudable increase in the percentage of laboratory primates that are housed socially^[32,33]^. The challenge in an active neuroscience lab is that the vivarium population is unstable. Animals are removed after variable periods of time and replaced by new animals, making it difficult to establish and maintain compatible pairs because the population is in flux. It has been reported that converting macaques from single-housing to pair-housing can be accomplished with a rate of serious injury of only 0.8%, but our success at forming new pairs has been far more limited^[34]^. The veterinary staff carefully assessed Clive and Jude and concluded that they could not be housed communally without a high risk of injury. The aggressive nature of macaques, especially males, is a practical reality that must be acknowledged because it explains why compatible social housing is so hard to achieve. At some National Primate Research Centers, animals are housed in large outdoor enclosures, considered the gold standard for maintaining macaques in captivity^[30]^. Despite such optimal conditions, animals are wounded in skirmishes between rivals on an occasional basis, requiring treatment by veterinary staff. Even macaques free in a natural environment inflict major injuries on each other (Fig. 7). Social housing reduces the risk of SIB, but it introduces a new risk, namely, potential injury from another animal.

Jude was given haloperidol and naltrexone to treat his SIB. There are small case series supporting the use of antipsychotics and opioid receptor blockers for the treatment of SIB in macaques^[7,35,36]^. These classes of medications have also been used to treat non-suicidal self-injury in humans, but data showing efficacy are weak^[37]^. Given that self-injury is common in domestic monkey colonies and among human adolescents, it is remarkable that little effort has been made to conduct randomized, masked clinical trials to validate pharmacotherapy. To date, no medication has been approved by the U.S. Food and Drug Administration for the specific purpose of treating non-suicidal self-injurious behavior. It should be stressed that to assess the response to a drug in a monkey, it must be given by intramuscular or subcutaneous injection if the animal rejects oral medications. Jude was able to recognize the bitter taste of haloperidol and naltrexone and dropped the treats containing these medications into his waste pan. Therefore, apparent treatment failure was actually a failure of drug delivery.

Efforts to manage Jude's SIB were hindered by difficulty agreeing upon its frequency. Initially, the LARC staff reported that video observation had captured one episode per min. This rate would correspond to 720 episodes during a 12-h lights-on cycle. Later, the LARC leadership cited a figure of over 50 episodes per day. Our video monitoring showed a rate of one episode per h, equating to 12 episodes per day. The episode we recorded lasted 11 s, suggesting that Jude engaged in SIB less than 0.5% of the time. It would have been optimal for all parties to review the video data to reconcile these discrepant measurements of the SIB rate.

It was also difficult to assess the severity of SIB because the monkey's fur made it difficult to observe injury to his limbs. To carry out an examination, the animal was anesthetized twice with ketamine. Going forward, there was concern that frequent exams would be necessary under anesthesia to monitor the animal's injuries. Shaving the monkey's wrists and ankles would have made it possible to detect injury without resorting to repeated exams under anesthesia. Whether fur removal affects the severity or frequency of SIB is unknown and would be a question of practical value to investigate through a controlled, prospective study. At the very least, it might decrease SIB in Category 2 by reducing the opportunity for hair plucking.

The United States Department of Agriculture supports scientific research by enforcing regulations governing animal welfare. An unintended consequence stemming from its oversight role is that institutions may be reticent to request veterinary advice from the agency. It would be desirable to create a channel, outside the auspices of regulatory enforcement, to encourage inquiries seeking guidance about animal care. Institutional leadership should also be careful not to prioritize risk avoidance over support for animal welfare^[38]^.

The veterinary literature contains little guidance regarding the category of SIB that constitutes a reason for euthanasia of a monkey. In 2001, there were an estimated 15,000 individually caged macaques in the United States, with potentially up to 3,000 exhibiting this behavior^[39]^. To euthanize all monkeys manifesting SIB would constitute an unacceptable waste of animal life. Caution is warranted when drawing conclusions from a single case, but we suggest that SIB corresponding only to Categories 4 and 5, as defined by the National Primate Research Centers, be considered a potential indication for euthanasia. Animals exhibiting SIB in Categories 1, 2, and 3, who may have behavioral abnormalities but only superficial wounds that do not require medical attention, usually can be managed without euthanasia. Efforts should be undertaken to modify their behavior by reducing visual isolation, attempting pair-housing, and administering medications that might be effective. Euthanasia should not be performed as a prophylactic tactic to prevent SIB from escalating from Category 3 to Category 4 or 5, because such progression in the severity of SIB occurs only rarely^[21]^. It might be useful for an expert consensus panel comprised of laboratory animal veterinarians, ethicists, and scientific investigators to establish guidelines for when euthanasia is appropriate in non-human primates exhibiting SIB.

This report has limitations inherent to any single case study, and the management of this animal's case may not be generalizable. The history was subject to a retrospective reconstruction of events, based on a detailed log of daily events and the record preserved by email exchanges. Access to the video data acquired by the LARC staff was limited, resulting in unreconciled estimates of SIB frequency. Finally, many individuals were involved in the care of this animal, and it is acknowledged that judgments made in good faith may differ.

## Conclusions

SIB in laboratory monkeys poses a difficult management challenge because the causes are not well understood and effective treatments are lacking. Social isolation was likely an instigating factor in the animal described in this report and should be avoided whenever possible. When SIB occurs, it should be graded according to the National Primate Research Center Scoring System. It is recommended that an expert consensus panel comprised of primate veterinarians, ethicists, and scientific investigators establish guidelines for SIB management, focusing in particular on the level of self-injury for which euthanasia is appropriate. Until a consensus is published, euthanasia is suggested only for monkeys that exhibit a moderate (Category 4) or severe (Category 5) level of SIB.

## Supplementary Material

Data availability

All data generated or analyzed during this study are included in the published article and its supplementary information files: https://osf.io/4gkz8/files/osfstorage/68b7782265113fae162c2615
